# Changes in the Trophic Pathways within the Microbial Food Web in the Global Warming Scenario: An Experimental Study in the Adriatic Sea

**DOI:** 10.3390/microorganisms8040510

**Published:** 2020-04-03

**Authors:** Mladen Šolić, Danijela Šantić, Stefanija Šestanović, Natalia Bojanić, Slaven Jozić, Marin Ordulj, Ana Vrdoljak Tomaš, Grozdan Kušpilić

**Affiliations:** 1 Institute of Oceanography and Fisheries, 21000 Split, Croatia; solic@izor.hr (M.Š.); sesta@izor.hr (S.Š.); bojanic@izor.hr (N.B.); sjozic@izor.hr (S.J.); ana.vrdoljak@izor.hr (A.V.T.); kuspe@izor.hr (G.K.); 2 Department of Marine Studies, University of Split, 21000 Split, Croatia; marin.ordulj@unist.hr

**Keywords:** microbial food web, global warming, sensitivity to temperature, trophic interactions, carbon flow, Adriatic Sea

## Abstract

A recent analysis of the Mediterranean Sea surface temperature showed significant annual warming. Since small picoplankton microorganisms play an important role in all major biogeochemical cycles, fluxes and processes occurring in marine systems (the changes at the base of the food web) as a response to human-induced temperature increase, could be amplified through the trophic chains and could also significantly affect different aspects of the structure and functioning of marine ecosystems. In this study, manipulative laboratory growth/grazing experiments were performed under in situ simulated conditions to study the structural and functional changes within the microbial food web after a 3 °C increase in temperature. The results show that a rise in temperature affects the changes in: (1) the growth and grazing rates of picoplankton, (2) their growth efficiency, (3) carrying capacities, (4) sensitivity of their production and grazing mortality to temperature, (5) satisfying protistan grazer carbon demands, (6) their preference in the selection of prey, (7) predator niche breadth and their overlap, (8) apparent uptake rates of nutrients, and (9) carbon biomass flow through the microbial food web. Furthermore, temperature affects the autotrophic and heterotrophic components of picoplankton in different ways.

## 1. Introduction

Marine microorganisms are an integral part of all major biogeochemical cycles, fluxes, and processes occurring in marine systems. Heterotrophic picoplankton (HPP; mostly heterotrophic bacteria) and autotrophic picoplankton (APP; *Prochlorococcus* (PROC), *Synechococcus* (SYN), and pico-eukaryotic algae (PE)) represent the major components of the marine picoplankton (PICO) community [1,2,3,4]. Heterotrophic bacteria (HB) play an important role in aquatic ecosystems through the assimilation of dissolved organic matter to sustain their metabolism and produce new biomass [5], as well as through the decomposition of organic matter and transformation of inorganic compounds into forms suitable for primary producers [6]. APP contributes considerably to carbon production (up to 90%) and energy flow [7,8]. In the Mediterranean Sea, the contribution of APP to primary production varies from 31% to 92%, and their impact seems to be more pronounced in oligotrophic waters [1]. Therefore, PICO production has important implications for the ecology of the microbial food web (MFW) and biogeochemical cycling in marine ecosystems [9]. All PICO groups are consumed by heterotrophic nanoflagellate (HNF) and ciliate (CIL) grazers, forming a link (‘microbial loop’) to higher trophic levels [10].

Additionally, the growth of PICO in aquatic systems is affected by environmental factors (e.g., light, temperature, etc.), nutrient availability, grazing, and viral lysis [11,12,13]. It is well-established that temperature significantly influences microbiological processes such as production [14], growth rate [15,16], and growth efficiency [17], as well as grazing on bacteria [9,18,19] and viral lysis [20,21,22,23]. However, these processes have shown different sensitivity to temperature increases, which ultimately determines how the microbial community will respond to warming [24,25]. The organisms at the lower levels of the food web are responsible for the strong bottom-up processes that ultimately control the structure and dynamics of the upper trophic levels [26]. Hence, it is possible that even minor changes at the base of the food web, as a response to human-induced temperature increase and/or nutrient input, can be amplified through trophic chains and can thus significantly affect different aspects of the structure and functioning of marine ecosystems (e.g., [27,28,29]). Therefore, investigating the impact of warming on microbial communities is necessary to gain a better understanding of the global carbon cycle in seawater.

Global and atmospheric climate change is altering the thermal conditions in the Adriatic Sea and, consequently, the marine ecosystem. The semi-enclosed nature of the Mediterranean Sea (including the Adriatic Sea) combined with reduced inertia, due to the relatively short residence time of its water masses, makes it highly reactive to external forces, in particular global warming [30]. A recent analysis of the Mediterranean Sea surface temperature showed significant annual warming (from 0.24 °C decade^−1^ west of the Strait of Gibraltar to 0.51 °C decade^−1^ over the Black Sea) [31]. Sea surface temperature (SST) along the eastern Adriatic coast increased by an average of 1.03 °C from 1979 to 2015, while a strong upward (almost linear) trend of 0.013 °C month^−1^ has been recorded since 2008 [32]. Global climatic models predict that water temperatures will increase by 2–4 °C, on average, over the next few decades [33]. There is no doubt that global climate change is altering the thermal conditions in the Adriatic Sea [34] and changing the functioning of the marine ecosystem [35,36]. Since a small increase in temperature can greatly alter the microbial role in the global carbon cycle, it is very important to understand the effect of warming on the MFW.

In this study, laboratory growth/grazing experiments were performed under in situ simulated conditions in order to study the structural and functional changes within the MFW after a 3 °C rise in temperature. The study involved: (i) HPP, which include two physiological groups of heterotrophic bacteria (HB) distinguished by flow cytometry, namely, high nucleic-acid bacteria (HNA) and low nucleic-acid bacteria (LNA); (ii) autotrophic picoplankton (APP) that includes two cyanobacteria (CB) groups, namely, *Prochlorococcus* (PROC) and *Synechococcus* (SYN) as well as pico-eukaryotic algae (PE); and (iii) protistan grazers, namely, heterotrophic nanoflagellates (HNF) and ciliates (CIL). The experiments were performed in April 2019 when water-column mixing transports nutrients to the surface layer. Previous results obtained in the Adriatic Sea [37] confirmed that a rise in temperature caused a significant increase in bacterial growth at temperatures below 16 °C and levelled off at higher temperatures, whereas the impact of temperature on APP was linear along the entire range of the investigated temperatures (from 10.5 °C to 23.6 °C) [38]. Therefore, we consider temperature range from 14 °C (ambient temperature) to 17 °C (after a 3 °C increase in temperature as an experimental manipulation) to be the most interesting for a more detailed study of changes that occur in the MFW.

Accordingly, the main aim of this study was to investigate the possible direction of changes in the MFW, in the global warming scenario. Specifically, how a 3 °C rise in temperature affects changes in: (1) the growth and grazing rates of the most important groups of picoplankton and their carbon biomass flow through the MFW; (2) the relationship between HNF and CIL predators in terms of their food niche breadth and their overlap, satisfying their carbon and their preference in the selection of prey; (3) apparent uptake rates of nutrients.

We hypothesized that the rise in temperature would affect the qualitative and/or quantitative changes of the majority of these parameters and that temperature would affect the autotrophic and heterotrophic components of picoplankton in different ways. The results could help to gain a better understanding of the possible changes in carbon transfer and energy flow under warming conditions, as well as to detect possible consequences for marine biogeochemical cycles.

## 2. Materials and Methods

All abbreviations used in this paper are in the table of Appendix A.

### 2.1. Growth/Grazing Experiment

Seawater was collected in April 2019 from Kaštela Bay (central Adriatic Sea; 43°31’ N; 16°22’ E) in order to set up a growth/grazing experiment that was performed in the laboratory under in situ simulated conditions. A total of 100 L of ambient seawater was collected from 1 m depth, transferred to the laboratory, and filtered immediately through 200 µm mesh to remove larger zooplankton predators. The experiment started two hours after seawater collection. The values of environmental parameters and ambient abundances (i.e., initial experimental abundances) of the studied microbial groups are listed in the Supplementary material (Table S1).

#### 2.1.1. Experimental Settings

Growth and grazing parameters were estimated using the size-fraction technique [39,40]. A 200 μm plankton net was used to remove large predators, a 10 μm pore size polycarbonate membrane was used to remove HNF predators (mostly ciliates, CIL), and a 2 μm pore size polycarbonate membrane was used to remove picoplankton (PICO) predators. To prevent cell breakage, the size-fraction <10 μm was filtered by gravity and <2 μm using a <2 kPa vacuum. The numbers of bacteria and PROC did not change after the passage through the 2 μm filter, whereas the number of SYN decreased by 5%–7%. The size fractionation <10 µm was chosen, based on previous studies at this site, to eliminate ciliates but not HNF [38]. The filtration process was designed to exclude picoplankton grazers from the 2 μm filtered fraction, allowing them to remain in the 10 μm fraction. We examined the influence of fractionation on HNF and found that about 3% to 8% of the HNF cells (very small pico-flagellates) passed through the 2 μm filters. Since their number did not change during the experiments, we assumed that these cells did not significantly affect picoplankton growth rates. This study did not include pigmented nanoflagellates, which could be also responsible for the grazing on picoplankton (mixotrophs). However, our unpublished data showed that heterotrophic nanoflagellates contributed much more in the total grazing in comparison to pigmented flagellates in the study area.

Each size fraction of a final volume of 4 L was then transferred to 5 L incubation bottles (run in triplicate), previously cleaned with 10% HCl and rinsed with Mili-Q water. Initially, all bottles were supplied with 5 μM nitrate (NaNO_3_) and 0.25 μM phosphate (KH_2_PO_4_). This manipulation ensured the exponential growth of all study groups over a three day period, reaching plateau stages, which enabled the determination of the maximum reached abundances, i.e., the carrying capacities (K).

Two identical experimental sets were incubated simultaneously in two thermostatic chambers at ambient temperature (14 °C) and 3 °C above ambient temperature (17 °C) for 96 h. In the experiments performed at 3 °C above in situ temperature, microorganisms had been exposed to a gradual rise in temperature during the 15 h period (temperature was increased by 0.2 °C every hour). The thermostats were equipped with six daylight lamps (LH-TS LED 18 W, 6000 K) that were programmed to follow the day–night cycle (constant light intensity) for the day of the year. Irradiance value during experiment was 53 μmol m^−2^ s^−1^, which is an optimum light treatment for photosynthetic picoplankton [41,42].

Additionally, at both temperatures, one set of bottles with a 2 μm fraction was simultaneously incubated in the dark. During the incubation process, the bottles were shaken gently. Subsamples for all analyses were taken at the beginning of the experiments and after 24, 48, 72, and 96 h. Triplicate subsamples for cell count were poured into sterile acid-washed glass bottles, fixed immediately, and frozen (–80 °C) until analysis (within 24 h after sampling).

#### 2.1.2. Determination of Growth and Grazing Parameters

Net growth rates (µ, day^−1^) of each microorganism group were calculated during the period of their exponential growth for each incubation bottle [43]:

(1) $$µ=\frac{\left(\ln N_{E}-\ln N_{B}\right)}{t}$$

where *N_B_* and *N_E_* represented the number of organisms at the beginning and at the end, respectively, of the period of exponential growth; and *t* is the duration of exponential growth in days.

Grazing rates (g) were calculated as the difference between growth rates in a predator-free fraction and growth rates in the presence of predators (for more details see Table 1).

Production rates (P, μg C L^−1^ day^−1^) and losses due to grazing (G, μg C L^−1^ day^−1^) were estimated using the following equations:

P = μ × B(2)

G = g × B(3)

where B is the cell biomass (μg C L^−1^) at sampling time.

#### 2.1.3. Removal of Prey Standing Stock and Production

The percentage of the prey standing stock (SSR) and prey production (PPR) removed daily by grazing was calculated according to James and Hall [44] and Safi et al. [45]:

(4) $$SSR\;\left(\%\right)=\left(1-e^{-g}\right)\times 100$$

(5) $$PPR\;\left(\%\right)=100\times \frac{\left(1-e^{-g}\right)}{\left(e^{\mu }-1\right)}$$

where *µ* is maximum growth rate and *g* is grazing rate.

#### 2.1.4. Ingestion Rate (I)

Number of ingested prey cells per predator cell was calculated as:

(6) $$I=g\times \frac{N_{PREY}}{N_{PREDATOR}}$$

where *g* is the grazing rate; and *N_PREY_* and *N_PREDATOR_* are geometric mean concentrations of prey and predator, respectively, during the experiment.

### 2.2. Bacterial Carbon Respiration, Carbon Demand, and Growth Efficiency

Bacterial carbon respiration (BR) was estimated from the consumption of dissolved oxygen in Biological Oxygen Demand (BOD) bottles, assuming a respiratory quotient of 1. Seawater filtered through 0.8 µm polycarbonate filters (Millipore, Millipore Merck KGaA, Darmstadt, Germany to remove phytoplankton and bacterial grazers was siphoned into 70 mL BOD bottles. For each treatment, BR was measured in triplicate at the beginning of the experiment and after 24, 48, 72, and 96 h. Oxygen concentrations were determined by Winkler titration using a potentiometric electrode. To estimate bacterial growth efficiency (BGE) and bacterial carbon demand (BCD), bacterial respiration (BR) was measured in parallel with bacterial production (BP), which was estimated as a difference of bacterial biomass between the beginning and the end of the incubation time. Bacterial carbon demand was estimated as BCD = BP + BR, and bacterial growth efficiency as BGE (%) = (BP/BCD) × 100.

### 2.3. HNF and Ciliate Gross Growth Efficiency (GGE)

HNF and ciliate gross growth efficiencies were calculated as:

GGE (%) = P/G × 100(7)

where P is HNF or ciliate production; and G is total HNF or ciliate grazing on all prey groups.

### 2.4. Calculation of Grazing Preference Index

To evaluate prey selection, the Manly–Chesson preference or selection index (alpha index, α) was calculated [46,47,48]:

(8) $$\alpha_{i}=\frac{d_{i}/e_{i}}{\sum_{i=1}^{m}d_{i}/e_{i}}$$

where d*_i_* is the proportion of prey item *i* in the diet; e*_i_* is the proportion of prey item *i* in the environment; and m is the number of prey items in the environment.

Since α values are normalized, they range from 0 (complete avoidance) to 1 (complete preference). If α = 1/m, the predator is feeding randomly, and the prey is consumed in proportion to its abundance in the environment; whereas α > 1/m indicates the preference and α < 1/m indicates the avoidance of prey consumption. 

### 2.5. Sensitivity to Temperature Analyses

The sensitivity of microbial production (P) and grazing on microbial prey groups (G) to temperature is displayed on an Arrhenius plot (widely applied graphical presentation of the Arrhenius law), which describes the relationship between temperature and biological reaction rates:

(9) $$\mathrm{Ln}\;BRR=\mathrm{Ln}\left(A\right)-\frac{E_{a}}{R}\;\frac{1}{T}$$

where *BRR* is studied biological reaction rate (P and G in this study); *A* is the theoretical *BRR* in the absence of *E_a_*; *E_a_* is activation energy (J mol^−1^); *R* is universal gas constant (8,314 J mol^−1^K^−1^); and *T* is temperature in Kelvin. Regression statistics for the Arrhenius plots are included in Supplementary Materials (Table S3).

### 2.6. Niche Breadth and Niche Overlap Measures

Some predators are more specialized than others and measures of niche breadth attempt to measure this quantitatively. Since the abundance of different microbial prey types differs significantly among the many ways of expressing niche breadth, we chose Smith’s [49] and Hurlbert’s [50] measures (for more details see Table S3 in Supplementary Materials). One way of understanding food web organization and possible competition between predators for sharing prey is the measurement of overlap in prey consumption among predators (food niche overlap). The two predator assemblages (nano- and micro-plankton) were distinguished by size without taking into consideration their taxonomic composition. In this study we compared Renkonen’s [51], Horn’s [52], and Hurlbert’s [50] indices of niche overlap (for more details see Table S3 in Supplementary Materials).

### 2.7. Data Analysis

#### 2.7.1. Environmental Parameters

Temperature and salinity were measured using CTD multiparameter probes (Idronaut and SeaBird) with >±0.01 °C and ±0.02 accuracy, respectively. Dissolved oxygen concentration was determined by Winkler titration [53]. Nutrients (NO_3_^−^, NO_2_^−^_,_ NH_4_^+^, and PO_4_^3−^) were analyzed using a Bran+Luebbe AutoAnalyser (II and III models) and applying standard colorimetric methods [54]. Daily apparent nutrient uptake or production rates were calculated as linear regressions of their concentrations during the incubation period.

#### 2.7.2. Flow Cytometry Analysis

For cell counting, samples were analyzed using a Beckman Coulter CytoFLEX cytometer (Indianapolis, Ind., USA with a fast flow rate of 60 µL min^−1^. Samples for autotrophic cells analysis (2 mL) were preserved in 0.5% glutaraldehyde, frozen at –80 °C, and stored until analysis. Samples for analysis of bacteria were preserved in 2% formaldehyde kept at 4 °C until analysis. Autotrophic cells were divided into three groups (two cyanobacteria (*Synechococcus* and *Prochlorococcus*) and picoeukaryotes), distinguished according to light scattering, cellular chlorophyll content, and phycoerythrin-rich cell signals [55]. Sybr Green-I-stained non-pigmented (heterotrophic) bacteria were determined according to Marie et al. [56]. According to the cellular nucleic acid content, the bacterial populations were divided into two sub-groups, HNA (High Nucleic Acid content) and LNA (Low Nucleic Acid content) bacteria. Abundances of Sybr Green-I-stained HNF were determined according to Christaki et al. [57].

#### 2.7.3. Ciliates

In order to remove large zooplankton organisms from the ciliate samples, the seawater was filtered through 200 µm mesh. Sample volumes of 2 L were sedimented [58] for 48 h in cylinders and decanted down to a volume of 200 mL. Prior to microscopic analysis, the volume was further reduced to 20 mL. Decanting was carried out using a vacuum pump and a slightly curved pipette that removed water from the surface. Microscopic analysis of samples was carried out in a glass chamber (76 × 47 × 6 mm) using inverted microscopes (Olympus IMT-2, Hamburg, Germany, equipped with phase contrast, at 200× and 400× magnification. The entire bottom of the sedimentation chamber was analyzed and abundance of non-loricate ciliates and tintinnids was expressed as number of cells per liter. Samples were fixed with acid Lugol’s solution (2% final concentration) and stored in the dark at 4 °C until counting (no longer than two weeks later). Ciliates were counted, distinguished into size classes and major taxonomic groups, and identified down to genus or species level where possible.

#### 2.7.4. Abundance to Biomass Conversion

The biomass of studied PICO groups was calculated using the following cell-to-carbon conversion factors: 20 fgC cell^−1^ for heterotrophic bacteria [59,60]; 36 fgC cell^−1^ for *Prochlorococcus* [61]; 255 fgC cell^−1^ for *Synechococcus* [61]; 2590 fgC cell^−1^ for pico-eukaryotic algae [61]; and 0.22 pgC µm^−3^ for HNF [62]. The HNF biovolume was estimated using the lengths and widths of flagellate cells. These measurements were performed under an Olympus BX51 epifluorescent microscope equipped with an XM10-IR camera. Ciliate cell sizes were measured on approximately 200 specimens from all samples, using an ocular micrometer, and converted into biovolumes by approximation to the nearest geometric shape from measurements of cell length and width. After measurement of the plasmatic body dimension of non-loricates, bio-volumes were converted into C biomass using 190 fgC μm^−3^ [63]. In addition, the biomass of tintinnids was calculated using the formula 444.5 pgC + (lorica volume in µm^3^ × 0.053 pgC) per cell, according to Verity and Langdon [64].

### 2.8. Statistical Analysis

Statistical operations were performed by STATISTICA 9.0 software. Data normality was assessed by applying the Shapiro–Wilk W normality test. Grazing preference (alpha) index, niche breadth measures, and niche overlap indices were calculated using Ecological Methodology Programs (version 7) by Charles J. Krebs.

## 3. Results

### 3.1. Impact of Temperature Rise on Microbial Growth and Grazing Parameters

**Figure 1:**
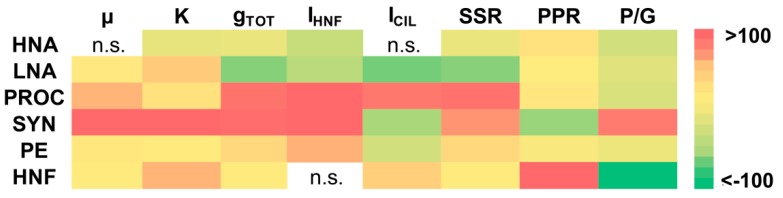
Heatmap of percentage changes of studied parameters after a 3 °C rise in temperature. µ, growth rate; K, carrying capacity; g_TOT_, total grazing rate; HNF, heterotrophic nanoflagellates; I_HNF_, HNF ingestion rate; I_CIL_, ciliate ingestion rate; SSR, prey standing stock removal; PPR, prey production removal; P/G, production/grazing ratio; HNA, high nucleic-acid bacteria; LNA, low nucleic-acid bacteria; PROC, *Prochlorococcus*; SYN, *Synechococcus*; PE, picoeukaryotes.

A review of growth and grazing parameters based on size-fraction experimental estimations is presented in Table S4 (Supplementary Materials), whereas a heatmap of percentage changes of studied parameters after a 3 °C rise in temperature is presented in Figure 1. Furthermore, temporal changes of biomass of studied groups during experiments were shown in Figure S1 (Supplementary Materials).

Maximal growth rates (predator-free condition) were higher than total grazing rates for all studied microbial groups, showing positive net growth rates. After a 3 °C rise in temperature (from initial ambient temperature of 14 °C to 17 °C), a statistically significant increase in growth rate was established for almost all microbial groups, with the exception of HNA. The highest increase in growth rates were observed for two cyanobacteria groups (SYN by 276% and PROC by 49%).

Carrying capacity (K), estimated as the maximum abundance reached at the *plateau* stage of the experiment, also increased with a rise in temperature in all groups except HNA (the greatest increases were measured for SYN by 105% and HNF by 49%). At ambient temperature, growth efficiencies of heterotrophic bacteria (HB = HNA + LNA; 41.6%) and HNF (48.6%) were higher than growth efficiency of CIL (22.5%). After a 3 °C rise in temperature, the growth efficiencies of HB, HNF, and CIL decreased to 22.7%, 40.1%, and 12.3%, respectively.

Total grazing rates (grazing in the 200 μm fraction includes all relevant predators, HNF and CIL in particular) reveal a statistically significant decrease with increasing temperature for heterotrophic prokaryotes (HNA and LNA groups), and a statistically significant increase for autotrophic picoplankton (APP) groups (SYN, PROC, and PE) and HNF. As with the growth rates, the highest increase in grazing rates was also observed for two cyanobacteria groups (SYN by 98% and PROC by 94%). This pattern was followed by the HNF ingestion rate (expressed as carbon biomass consumed per HNF cell), evidenced as an increase in the consumption of APP (particularly SYN and PROC whose consumption increased by 333% and 137%, respectively) and as a decrease in consumption of heterotrophic prokaryotes (HNA and LNA), after a 3 °C rise in temperature. Likewise, the ingestion rate of ciliates showed an increase in consumption of PROC (by 91%) and HNF (by 31%), and a decrease in consumption of LNA (by 65%), SYN (by 41%), and PE (by 19%) after the rise in temperature.

**Figure 2:**
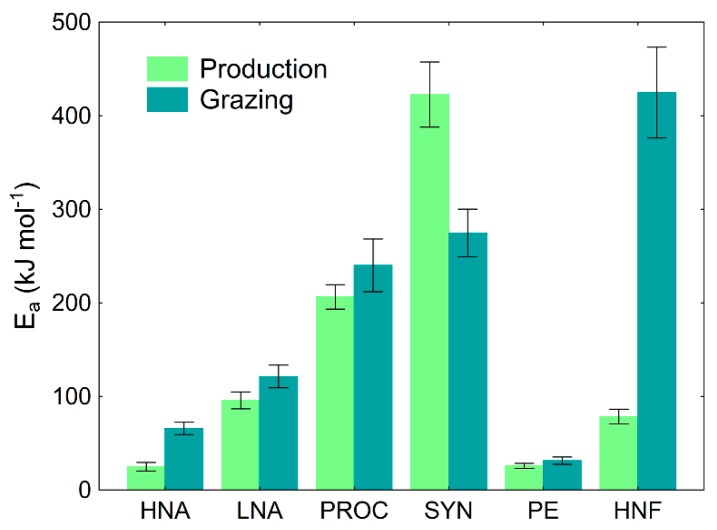
Apparent activation energy (E_a_) as mean ± standard deviation (SD) was derived from the regression slope of the Arrhenius plots for production and losses-to-grazing of the studied microbial groups. HNA, high nucleic-acid bacteria; LNA, low nucleic-acid bacteria; PROC, *Prochlorococcus*; SYN, *Synechococcus*; PE, picoeukaryotes; HNF, heterotrophic nanoflagellates).

In warming conditions, relative standing stock removal (SSR%) decreased for heterotrophic prokaryotes (HNA and LNA) and increased for all APP groups (PROC, SYN, and PE), particularly due to HNF grazing (Figure 1; Table S2). Relative removal of prey production (PPR%) was higher for APP groups than for heterotrophic ones. After the rise in temperature, PPR increased for all groups, meaning a higher sensitivity of grazing mortality than production to warming, with the exception of SYN (see also Figure 2). Removal of SYN production significantly decreased in warming conditions, mostly because of a marked increase in SYN production, which was not accompanied by an equal increase in its grazing. These results are corroborated by the production/grazing ratio (P/G), which decreased in warming conditions for all groups (particularly for HNF), except for SYN which increased by 89%.

### 3.2. The Sensitivity of Microbial Production and Losses-to-Grazing to Temperature

Arrhenius plots show a statistically significant positive effect of the increase in temperature on production and losses-to-grazing for all studied microbial groups. Apparent activation energy (E_a_) derived from the regression slope of the Arrhenius plots (see Table S2 in Supplementary Materials), as a measure of sensitivity of production and grazing rates to temperature showed the highest sensitivity for PROC and SYN production and losses-to-grazing to temperature compared to all other picoplankton groups (Figure 2).

Furthermore, HNF losses-to-grazing also showed very high sensitivity to grazing. In addition, this analysis showed higher sensitivity of grazing mortality than production to temperature for all groups except SYN.

### 3.3. Relative Contribution of Prey Biomass to Total Biomass Ingested by Protistan Predators

**Figure 3:**
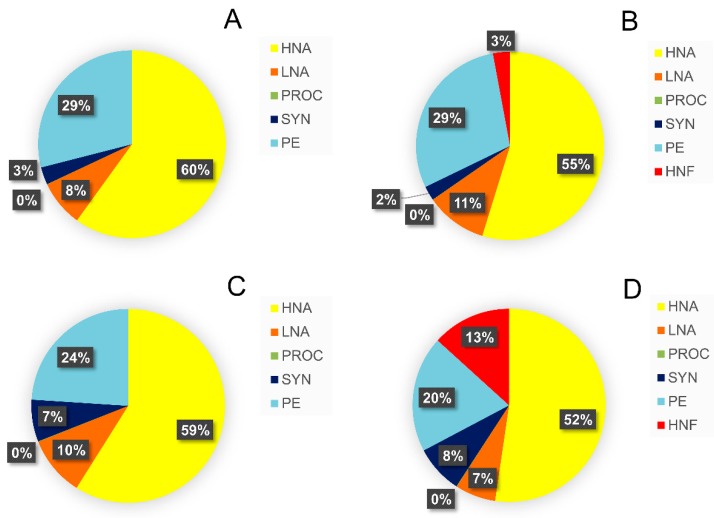
Relative contribution of prey biomass to total biomass ingested by HNF (**A**,**C**) and ciliates (**B**,**D**) at ambient temperature of 14 °C (**A**,**B**) and after a 3 °C rise above ambient temperature (**C**,**D**). HNA, high nucleic-acid bacteria; LNA, low nucleic-acid bacteria; PROC, *Prochlorococcus*; SYN, *Synechococcus*; PE, picoeukaryotes; HNF, heterotrophic nanoflagellates.

In general, the largest portion of picoplankton biomass ingested by HNF and CIL came from HNA (from 50% to 60%) and PE (from 20% to 30%), whereas the contribution of PROC to total ingested biomass was negligible (less than 0.1%; Figure 3).

The most significant change in HNF grazing, that occurred after a 3 °C rise in temperature, was increased proportion of ingested SYN biomass (from 3% to 7%), mostly at the expense of the PE which decreased from 29% to 24% in terms of contribution in total ingested biomass Even more noticeable changes after a rise in temperature occurred in ciliate grazing pressure. The contribution of HNF and SYN in satisfying CIL carbon demand increased by 10% and 6%, respectively (Figure 3).

### 3.4. Analysis of Prey Preference by Protistan Predators

**Figure 4:**
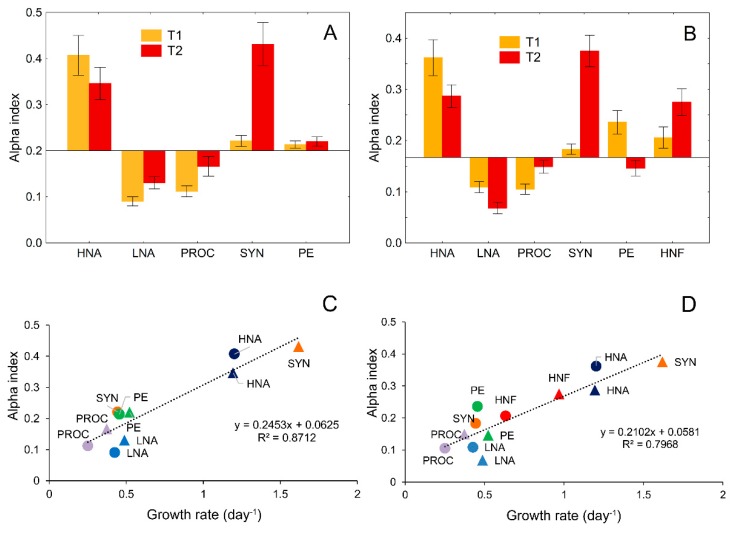
The Manly–Chesson normalized alpha index (mean ± SD) showing a preference or prey selection for HNF (**A**) and ciliate grazing (**B**) at ambient temperature (14 °C; T1) and after a 3 °C rise above ambient temperature (T2). Regression of the prey preference (expressed as alpha index) of HNF (**C**) and ciliates (**D**) to microbial growth rates. Circles represent ambient temperature, and triangles represent 3 °C above ambient temperature. Colors in panels C and D serves to distinguish species pairs at two temperatures (in C and D, standard deviations of the alpha index are the same as in A and B). HNA, high nucleic-acid bacteria; LNA, low nucleic-acid bacteria; PROC, *Prochlorococcus*; SYN, *Synechococcus*; PE, picoeukaryotes; HNF, heterotrophic nanoflagellates).

The results of the normalized Manly–Chesson preference or selection index (alpha index) showed that HNF consumed a higher proportion of HNA and a lower proportion of LNA and PROC than their proportion in the environment at ambient temperature (14 °C; Figure 4A). After a 3 °C rise in temperature, besides HNA, HNF showed a strong preference for SYN as well.

Ciliates also showed a strong preference for HNA, while the affinity for PE and HNF was of slightly less intensity at ambient temperature. After the experimental rise in temperature, the preference for SYN and HNF increased markedly, mostly at the expense of HNA, although they still preferred them. On the other hand, ciliates avoided PE completely (Figure 4B). A statistically significant relationship between growth rates and alpha index suggested that predator preference to particular prey was not or not exclusively related to their abundances/biomasses, but to their growth rates, i.e., relative changes in their production (Figure 4C,D).

### 3.5. Food-Niche Relationship among Protistan Predators

**Figure 5:**
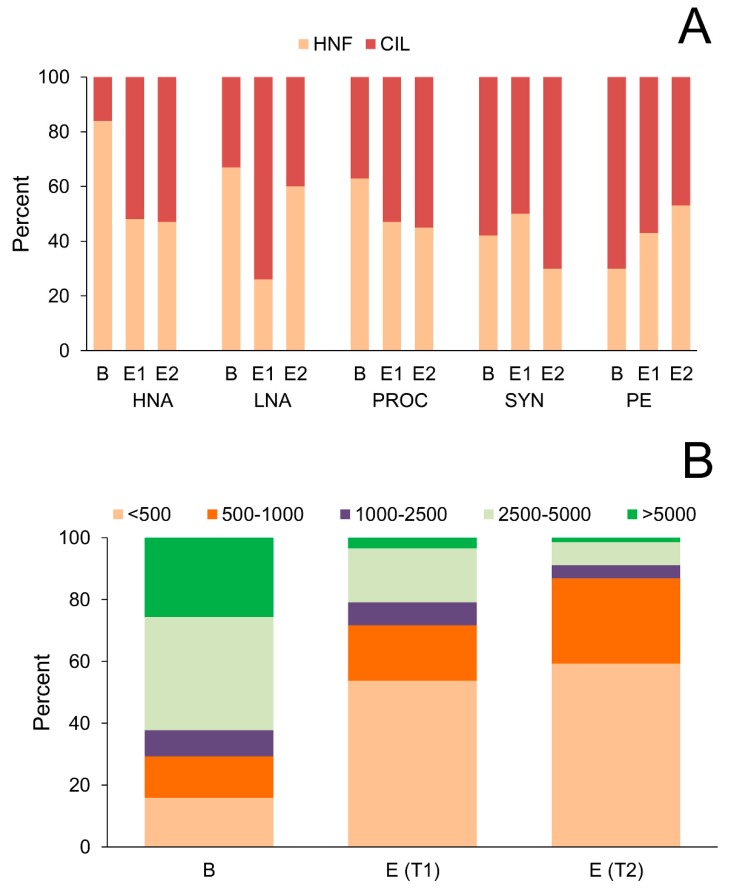
(**A**) Relative contribution of heterotrophic nanoflagellates (HNF) and ciliates (CIL) to total grazing of picoplankton groups at the beginning of the log phase of experiment (B) and at the end of the log phase of the experiment at ambient temperature (E1) and at the elevated temperature (3 °C increase; E2). (**B**) Changes of ciliate cell size categories during the experiment. Ciliate size fractions are expressed as carbon biomass per cell (pgC cell^−1^). HNA, high nucleic-acid bacteria; LNA, low nucleic-acid bacteria; PROC, *Prochlorococcus*; SYN, *Synechococcus*; PE, picoeukaryotes.

Considering PICO biomasses overall (HNA+LNA+PROC+SYN+PE), 45% of that biomass was consumed by HNF and as much as 55% by CIL, at ambient temperature. After the rise in temperature, these values changed to 49% and 51%, consumed by HNF and CIL, respectively. These shifts are also evident in the composition of the prey. In warm conditions, the contribution of HNF to total grazing increased for LNA and PE, and decreased for SYN, whereas CIL grazing pressure showed the opposite pattern (Figure 5A).

Furthermore, the relative contribution of HNF and CIL to total grazing of PICO groups changed during the experiment. A comparison between the first period of the experiment (beginning of log phase) and the end of the experiment (end of log phase before reaching the lag phase) showed that the relative contribution of CIL to total grazing, increased for groups with smaller cells (HNA, LNA, PROC; Figure 5A). The relative increase of CIL grazing pressure on small cells during the experiment was accompanied with changes in cell size structure of the CIL community. Two smallest CIL size fractions (<500 and 500–1000 pgC cell^−1^) accounted for 50% of the total number of CIL at the beginning of the log phase. During the experiment, their share to CIL biomass increased to 72% and 87% at ambient temperature and the 3 °C elevated temperature, respectively (Figure 5B).

Since the number of prey types was specified in this study, we did not focus on niche breadth and niche overlap (that were high, as expected) per se, but on the relative changes of these parameters after a rise in temperature, as well as between the beginning and the end of the log phase of growth (in the case of niche overlap). The values of Smith’s and Hurlbert’s measure of niche breadth increased with the rise in temperature for both HNF and CIL predators (Table 2). This pointed to the greater evenness in the consumption of different types of prey (see also Figure 3).

Furthermore, during the experiment, food-niche overlap between HNF and CIL increased, mostly because of change in the size structure of the CIL community towards smaller sized fractions. On the other hand, the rise in temperature reduced food-niche overlap between HNF and CIL, mostly because of the marked increase of HNF contribution to total biomass ingested by CIL (Table 2). In general, competition for picoplankton prey between HNF and CIL was strong, but slightly lower in conditions of elevated temperature.

### 3.6. Relationship between Nutrient Uptake Rate and Changes in the Contribution of Different Microbial Groups to Total Picoplankton Biomass and Production

**Figure 6:**
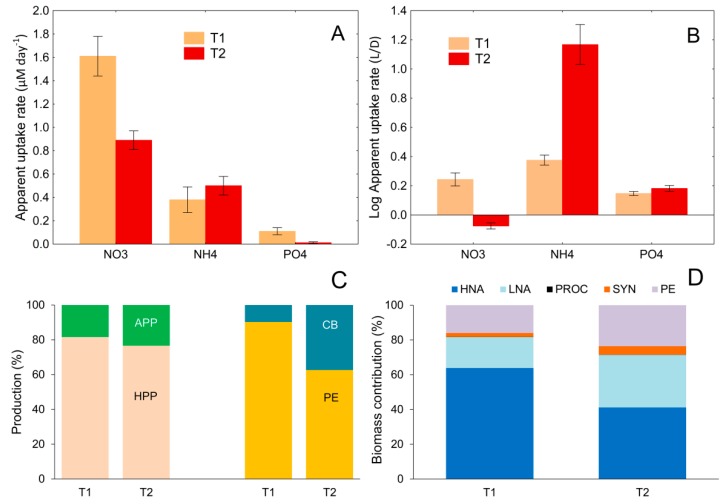
Apparent uptake rate (mean ± SD) of NO_3_^−^, NH_4_^+^, and PO_4_^3−^ (**A**), ratios of apparent uptake rates between light and dark conditions (L/D) in 2 μm fraction (**B**), contribution of APP and HPP to total PICO production, and contribution of cyanobacteria (CB) and PE to total autotrophic picoplankton (APP) production (**C**), and biomass contribution (%) of HNA, LNA, PROC, SYN, and PE to total picoplankton biomass (**D**). All are at ambient temperature (T1) and after a 3 °C rise in temperature (T2).

After a 3 °C rise in temperature, the apparent uptake of NO_3_^−^ and PO_4_^3−^ decreased, while the uptake of NH_4_^+^ increased (Figure 6A).

A comparison of nutrient consumption in 2 μm fraction, under light and dark conditions, showed that a significantly higher increase in NH_4_^+^ uptake, after the rise in temperature, occurred under light than under dark conditions, suggesting that NH_4_^+^ was dominantly consumed by an autotrophic component of picoplankton (Figure 6B). This is supported by the fact that in the warm conditions, the contribution of APP production to total picoplankton production (HPP+APP) increased from 18% to 24%, mostly due to a marked increase in the contribution of CB production (dominantly SYN) to total APP production (increased from 9% to 37%; Figure 6C). Furthermore, at the elevated temperature, the relative biomass of APP groups and LNA bacteria increased at the expense of HNA bacteria (Figure 6D).

### 3.7. Absolute and Relative Changes in Carbon Biomass Flux in Warming Conditions

**Figure 7:**
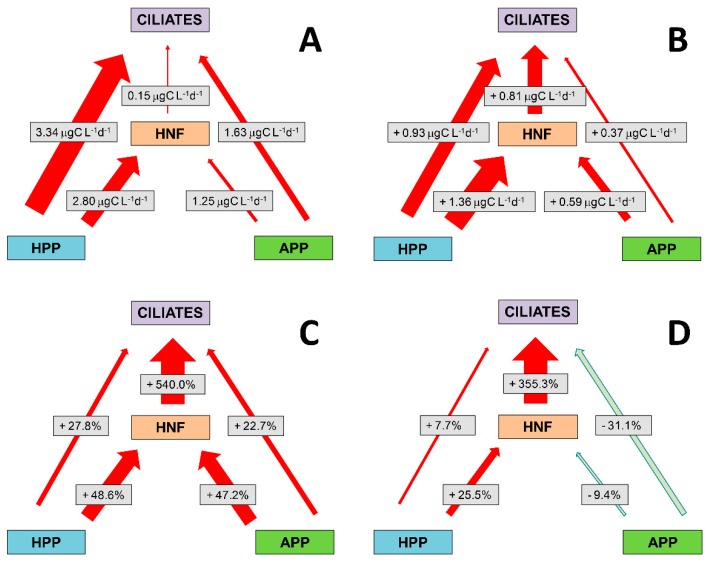
Absolute carbon biomass flow within the microbial food web at ambient temperature (**A**) and changes in flows after a 3 °C rise in temperature, expressed in three different ways: absolute changes in the amount of consumed carbon biomass (**B**); (ii) relative changes in the amount of consumed biomass (**C**); and relative changes in biomass grazing expressed as a percentage of prey production (**D**). Arrow thickness represents the relative importance of a particular carbon flow.

A schematic review of the carbon biomass flow within the microbial food web is shown in Figure 7. Picoplankton prey was divided into two groups, heterotrophic picoplankton (HPP = HNA + LNA) and autotrophic picoplankton (APP = PROC + SYN + PE). At ambient temperature, the dominant pathway of the carbon flow was from HPP toward NF and CIL, and to a lesser extent from APP to CIL (Figure 7A). Changes in flows after a 3 °C rise in temperature are presented in three different ways (Figure 7B–D). Absolute changes in the amount of consumed carbon biomass showed that the already high biomass flux from HPP to HNF and CIL increased further (particularly towards HNF) with a simultaneous increase of the carbon flux from HNF to CIL. Thus, the part of the direct trophic interaction between HPP and CIL was switched to an indirect route via HNF (Figure 7B). Similarly, the dominant pathway of direct APP biomass flux towards CIL also switched to an indirect pathway through HNF.

The HPP+APP → HNF → CIL flux is even more evident as a dominant pathway when the increase in consumed biomass is shown relatively (Figure 7C). The HPP and APP biomass fluxes towards HNF increased by almost 50%, and from HNF towards CIL by 540% after a 3 °C rise in temperature, and this was accompanied by a marked increase in HNF production (about 40%). Finally, when grazing was expressed as a percentage of production, the values increased for HPP, but decreased for APP suggesting a higher increase in grazing compared to production for HPP and vice versa for APP (particularly due to SYN, see also P/G ratio in Figure 1; Table S2), after the rise in temperature (Figure 7D). The highest relative increase (about 355%) in grazed production was established for HNF.

## 4. Discussion

Under non-limited nutrient conditions, picoplankton biomass was dominated by HNA (64%), followed by LNA (17%), PE (16%), SYN (3%), and PROC (<1%). Such picoplankton biomass distribution is in accordance with HNA (within heterotrophic prokaryotes) and PE (within autotrophic picoplankton) dominance in coastal regions, particularly during a spring period with high supply of nutrients. On the other hand, LNA bacteria and CB (particularly PROC) preferred oligotrophic waters [4,65,66,67,68], as they are better adapted to oligotrophic conditions [69,70]. An almost identical biomass distribution for picoplankton groups was found in the Galician coastal upwelling system as follows: HNA (55%), LNA (21%), PE (11%), SYN (6%), and PROC (1%) [71].

In general, after a 3 °C rise in temperature, the growth rate increased for all studied microbial groups with, however, quantitatively different responses depending on the group. Negative relationships with temperature were found for the HNA to LNA biomass ratio (after a 3 °C rise in temperature, this ratio decreased from 3.6 to 1.4), PE to CB biomass ratio (from 6.9 to 4.7), and for the HPP to APP biomass ratio (from 4.5 to 2.5). A similar relationship between the PE to CB ratio and temperature has also been reported by Otero-Ferrer et al. [71], however, contrary to our study, these authors found that temperature was negatively correlated with the contribution of LNA bacteria to heterotrophic biomass and with the contribution of APP biomass to total PICO biomass.

A reverse relation of carrying capacity K with temperature in HNA (negative relationship) compared to LNA (positive relationship), as well as correlated changes of growth rate and K*s* with temperature are supported by the study of Huete-Stauffer et al. [68]. Higher growth rates with increasing temperature also resulted in higher K, which is achieved mostly a day earlier (not shown), probably due to non-limited nutrient conditions [72], and this was strongly noticeable in SYN. Still, this result is not in accordance with the metabolic theory of ecology (MTE), which predicts that population density should decrease with increasing temperatures [73,74].

Within the HPP group, at ambient temperature, LNA showed a much lower growth rate compared to HNA. However, LNA were more sensitive to temperature rises, as also confirmed by the results of Morán et al. [75]. Studies have shown that LNA prefer warm waters [68,76]. Calvo-Díaz et al. [77] suggested that temperature plays a fundamental role during nutrient-rich environmental conditions and has little or no effect on bacterial growth when resource supply is low. Our previous study carried out in the coastal and open Adriatic Sea waters showed much higher sensitivity of bacterial growth to temperature at temperatures below 16 °C compared to higher temperatures, and slightly higher sensitivity to temperature in non-limited phosphorus conditions than in limited phosphorus conditions [37] (this study focused on phosphorus as a generally limited nutrient in the Adriatic Sea).

Compared to HB (both HNA and LNA), CB (PROC and SYN) were much more sensitive to temperature (both production and grazing), suggesting that heterotrophic as well as autotrophic picoplankton components respond differently to temperature rises. Contrary to HB, where the impact of temperature was more pronounced at temperatures below 16 °C and levelled off at higher temperatures, this impact on APP (especially CB) was linear along the entire range of investigated temperatures (from 10 °C to 26 °C) [38]. Responses of HPP production to temperature could be explained by nutrient-rich conditions during the experiment, sufficient to satisfy the higher HPP energy demands at higher temperatures. This is supported by Morán et al. [78] who revealed that a rise in temperature stimulates bacterial growth only in conditions of sufficient nutrients. However, our previous study on the interaction between temperature and phosphorus in controlling the picoplankton carbon flux indicated that a rise in temperature has a positive effect on all picoplankton groups, especially APP [38]. This is consistent with a temperature-modulated substrate affinity model [79], which suggests that available nutrients can be utilized better at higher temperatures.

The difference in temperature sensitivity between HPP and APP (especially CB) is in accordance with the idea that planktonic autotrophs and heterotrophs are characterized by different activation energies [80]. Moreover, there is general agreement that a rise in temperature increases the domination of pico-phytoplankton in total phytoplankton biomass and production [81,82,83]. Van de Poll et al. [84] reported that phytoplankton cell size decreased in summer while the contribution of cyanobacteria to water column productivity increased with temperature. Therefore, in the source utilization processes, CB are more sensitive to temperature increases, and in the global warming scenario an extremely important role in the microbial carbon flux could be expected. This is supported by the results of a multiannual study (2008–2015) carried out in the central Adriatic Sea, that reported continuous and significant rises in surface seawater temperature that was accompanied by significant increase in CB (PROC and SYN) and HNF abundances, but a decrease in HB abundances [32].

Sensitivity of grazing and growth rates to temperature showed similar patterns, with the exception of extremely high sensitivity of grazing on HNF to temperature. However, the data analysis showed consistent higher sensitivity of grazing mortality than production to temperature in all groups except SYN. They showed a marked increase in production at the elevated temperature that was not accompanied by an equal increase in grazing pressure. Our results for HPP (HNA and LNA) are not in accordance with the study of Sarmento et al. [9], who found that bacterial production increased at higher rates than bacterial losses to grazing. On the other hand, our results for PROC and PE are supported by several studies reporting that the mortality rate of autotrophs showed higher sensitivity to warming compared to their growth rate [24,85,86,87].

A positive effect of temperature on prokaryotic grazing rates and consequently an increase in their biomass transfer to higher trophic levels has been reported [87,88]. After a 3 °C rise in temperature, a significant increase in HNF grazing on CB (PROC and SYN), as well as in CIL grazing on HNF was found. This study suggests that CIL are important predators of not only HNF and picoplankton groups with larger cells (SYN and PE), but also picoplankton groups with smaller cells (HNA, LNA, and PROC). This is consistent with studies reporting a higher impact of microzooplankton than HNF on PICO prey stocks [89,90]. CIL can exert powerful control on the PICO community, both through direct grazing and trophic cascading [91,92].

The relative contribution of different types of prey to total PICO biomass ingested by predators was mainly related to prey growth rate rather than their abundance/biomass. Moreover, during the experiment, the community size structure of CIL changed in order to adapt better to the consumption of faster-growing picoplankton groups with smaller cells. This emphasizes that apart from abundances, it is also important to know the community structure of ciliates, e.g., microzooplankton assemblages [93]. The taxonomic composition of the ciliate community indicates non-loricate ciliates as the main consumers of PICO biomass at ambient temperature. After the experimental temperature rise, ciliates with lower than 1000 pgC cell^−1^ biomass, such as *Lohmanniella oviformis* and *Strombidium* sp., and small tintinnids, such as as *Stenosemella nivalis* and *Tintinnopsis nana*, became dominant in the ciliate community. The preferred prey size of CIL is positively related to oral diameter and is estimated at about 20% of lorica diameter [94]. A shift from larger to smaller ciliates coinciding with a shift from larger to smaller producers has also been reported [95,96]. Therefore, small CIL size fractions could be considered as major predators of PICO.

In warming conditions, both HNF and CIL switched their preference to SYN, a PICO component that showed the highest increase in growth rate. In such temperature conditions, considerable increase in CIL preference for HNF was also established. This is consistent with the results of Stoecker and Capuzzo [97] who found that ciliates feed preferentially on HNF and other larger cells. This reaffirms the importance of knowing the composition of the CIL community for gaining a better understanding of trophic relations within the microbial food web.

Obviously, trophic interactions between HNF and CIL in the marine environment are complex. Since, both plankton groups are sympatric predators of picoplankton prey, they are expected to be competitors for picoplankton prey [98]. However, at the same time, they form a predator–prey system. Moreover, due to their wide range of sizes (particularly within microzooplankton), predator–prey relationships are also possible within each of these groups [93,99]. In general, our study suggests strong competition for picoplankton prey between HNF and CIL. During the course of the experiment, the overlap of food-niches between HNF and CIL increased and reached a maximum at the end of the log phase, mainly due to a change in the size structure of the CIL community towards smaller size fractions. However, the overlap of niches (competition interaction) under warming conditions was reduced, mostly because of the marked increase in the HNF growth rate, which was accompanied by an increasing contribution of HNF to the total biomass ingested by CIL.

After the rise in temperature, the apparent uptake of NO_3_^-^ and PO_4_^3-^ decreased, while the uptake of NH_4_^+^ increased. This could be explained primarily by APP consumption, accompanied by an increase in APP production and their relative contribution to total picoplankton biomass. Increased concentration of NH_4_^+^ may influence the preference of phytoplankton for NH_4_^+^ vs. NO_3_^-^, mainly favoring APP production [100]. This implies NH_4_^+^ regeneration during the experiment as a possible consequence of bacterial regeneration, viral lysis, and/or excretion of predators. Bacterial regeneration of NH_4_^+^ occurred in low DOC/DON conditions, which determine net bacterial excretion rather than NH_4_^+^ uptake [101]. Such conditions were found during the spring in the Adriatic Sea [Šolić et al., unpublished].

Changes in the NH_4_^+^/NO_3_^−^ ratio showed a very strong positive relationship with APP [102]. Wafar et al. [103] found that N uptake by APP was mainly supported by regenerated N originating from NH_4_^+^ (66%) and urea (17%). A strong preference of APP for a reduced form of nitrogen (NH_4_^+^) [104,105] is particularly expressed in autotrophic prokaryotes (PROC and SYN). Thus, Moore et al. [106] reported that nearly all PROC and SYN isolates are limited to NH_4_^+^ as their source of nitrogen. Furthermore, Casey et al. [107] found that <10% of PROC populations used NO_3_^-^ as their source of N, despite their genetic capability to utilize NO_3_^−^ [108]. The study of Flombaum et al. [109] indicates that PROC and SYN abundance distributions over the major ocean regions are controlled by temperature and photosynthetically active radiation, discarding the role of NO_3_^-^ concentration. Moreover, the ratio of prokaryotic to pico-eukaryotic photoautotrophic biomasses in the north–western Mediterranean Sea decreased with increasing NO_3_^-^ supply [110], suggesting the ability of prokaryotic autotrophs to survive under nutrient starving conditions [111]. Furthermore, Otero-Ferrer et al. [71] reported negative relationship of SYN and LNA with NO_3_^−^ supply, whereas a unimodal function was shown for PE.

In general, after an experimental temperature rise of 3 °C, the proportion of picoplankton biomass channeled through the microbial food web towards the higher trophic levels increased. Similar results were found by several other studies [19,22,86]. APP showed higher relative increase in carbon flux towards predators compared to HPP. Incorporation of picoplankton carbon biomass into CIL occurs in two ways: via direct grazing on PICO and indirectly through grazing on HNF. The relative importance of the first pathway diminished, while the indirect pathway, through HNF, increased with temperature. The efficiency of PICO carbon transfer to the CIL depends on the contribution of CIL to total grazing on PICO. Thus, if PICO are mainly consumed by CIL, the efficiency of PICO biomass transfer will be higher than if PICO are mostly consumed by HNF when efficiency of transfer towards CIL is greatly reduced and the vertical flux of PICO biomass is slower. However, this study showed that although most of the PICO carbon biomass incorporated in CIL previously passed through HNF, the total amount of PICO carbon transferred to CIL was significantly higher at higher temperatures. Hence, in the global warming scenario, an increase of the picoplankton carbon flux towards higher trophic levels could be expected. Moreover, the role of APP in the carbon flux could become more significant.

## Figures and Tables

**Table 1:** Determination of growth and grazing parameters in the size-fraction grazing experiments. Picoplankton (PICO) include three components: heterotrophic bacteria, Prochlorococcus, and Synechococcus. µ(2µm), µ(10µm), µ(200µm) are the growth rates in <2 µm, <10 µm, and <200 µm size fraction, respectively. HNF, heterotrophic nanoflagellates; CIL, ciliates; gPICO(TOT), total grazing rate on PICO groups; gPICO(HNF), HNF grazing rate on PICO groups; gPICO(CIL), ciliate grazing rate on PICO groups; gHNF(CIL), ciliate grazing rate on HNF.

Experimental Settings	Size Fractions		
	2 µm	10 µm	200 µm
Prey organisms	PICO groups	PICO groups	PICO groups, NF
Predator organisms	No predators	HNF	HNF, CIL
Estimated growth rates	Maximal growth of PICO [µ_PICO(2µm)_]	Net growth of PICO [µ_PP(10µm)_] Maximal growth of HNF [µ_HNF(10µm)_]	Net growth of PICO [µ_PICO(200µm)_] Net growth of HNF [µ_HNF(200µm)_]
Estimated grazing rates	No grazing	HNF grazing on PICO: g_PICO(HNF)_ = µ_PICO(2µm)_ – µ_PICO(10µm)_	Total grazing on HNF: g_PICO(TOT)_ = µ_PICO(2µm)_ − µ_PICO(200µm)_ Ciliate grazing on HNF: g_HNF(CIL)_ = µ_HNF(10µm)_ − µ_HNF(200µm)_

**Table 2:** Food-niche breadth of heterotrophic nanoflagellate (HNF) and ciliate (CIL) predators and food-niche overlap between them.

Food-Niche Characteristic Title	Ambient Temperature		Ambient Temperature + 3 °C	
	HNF	CIL	HNF	CIL
Niche breadth: Smith’s measure (95% conf. limits)	0.943 (0.928–0.957)	0.881 (0.860–0.901)	0.972 (0.960–0.981)	0.939 (0.923–0.953)
Hurlbert’s standardized measure (95% conf. limits)	0.749 (0.706–0.792)	0.642 (0.606–0.677)	0.832 (0.784–0.880)	0.673 (0.620–0.725)
Niche overlap: Percentage overlap (%)	90.9		82.7	
Horn’s index	0.984		0.918	
Hurlbert’s index	1.666		1.423	

## Supplementary Materials

The following are available online at https://www.mdpi.com/2076-2607/8/4/510/s1, Table S1. Environmental parameter values and initial (ambient) abundances of studied plankton components. Table S2. Regression statistics for the Arrhenius plots showing the relationship of temperature (1/Kelvin × 1000) and the natural logarithm of production and grazing. E_a_—values of activation energy, where R is universal gas constant (8.314 J mol^−1^ K^−1^); *R*^2^, coefficient of determination; *p,* significance level of the regression analyses (ANOVA). Table S3. Review of niche breadth and niche overlap measures. Table S4. Mean ± standard deviation of growth rate (µ), carrying capacity (K), growth efficiency, total grazing rate (g_TOT_), HNF ingestion rate (I_HNF_), ciliate ingestion rate (I_CIL_), prey standing stock removal (SSR), prey production removal (PPR), and production/grazing ratio (P/G) for the studied microbial groups. HNA, high nucleic-acid bacteria; LNA, low nucleic-acid bacteria; HB, heterotrophic bacteria = HNA+LNA; PROC, *Prochlorococcus*; SYN, *Synechococcus*; PE, picoeukaryotes; HNF, heterotrophic nanoflagellates; CIL, ciliates) at ambient temperature (T1) and 3 °C elevated temperature (T2). The differences of studied parameters between two temperatures were tested with *t*-test for dependent samples. Percentage changes in parameter values in increased temperature conditions (T2) compared to initial ambient temperature (T1) were noted in the last column (positive changes are bolded). Figure S1. Biomass changes throughout experiments. A, high nucleic-acid bacteria; B, low nucleic-acid bacteria; C, *Prochlorococcus*; D, *Synechococcus*; E, picoeukaryotes; F, heterotrophic nanoflagellates. Full lines represent ambient temperature and dashed lines –3 °C elevated temperature.

## Appendix A. Table of Abbreviations

**Abbreviation**	**Full Term**
HPP	Heterotrophic picoplankton
APP	Autotrophic picoplankton
PROC	*Prochlorococcus*
SYN	*Synechococcus*
PE	Picoeukaryotes
PICO	Picoplankton
HB	Heterotrophic bacteria
MFW	Microbial Food Web
HNF	Heterotrophic nanoflagellates
CIL	Ciliates
HNA	High Nucleic Acid content bacterial group
LNA	Low Nucleic Acid content bacterial group
APP	Autotrophic picoplankton
CB	Cyanobacteria
SST	Sea surface temperature
K (cells mL^−1^)	Carrying capacity
µ (day^−1^)	Net growth rate
g (day^−1^)	Grazing rate
P (µg C L^−1^ day^−1^)	Production rate
G (µg C L^−1^ day^−1^)	Grazing
B	Cell biomass
I	Ingestion rate
BR	Bacterial carbon respiration
BOD	Biological Oxygen demand
BGE	Bacterial growth efficiency
BCD	Bacterial carbon demand
BR	Bacterial respiration
BP	Bacterial production
GGE	Gross growth efficiency
R	Universal gas constant (8314 J mol^−1^K^−1^)
SSR (%)	Standing Stock Removal
PPR (%)	Prey Production Removal
P/G	Production/Grazing ratio
E_a_	Apparent activation energy
T1 (°C)	Ambient temperature
T2 (°C)	3 °C elevated temperature
g_TOT_ (day^−1^)	Total grazing rate
I_HNF_ (pgC HNF^−1^ day^−1^)	HNF ingestion rate
I_CIL_ (ngC CIL^−1^ day^−1^)	Ciliate ingestion rate
(E_a_)	Apparent activation energy
DOC	Dissolved Organic Carbon
DON	Dissolved Organic Nitrogen
