# Supplementary material for: Changes in the Trophic Pathways within the Microbial Food Web in the Global Warming Scenario: An Experimental Study in the Adriatic Sea
Source: Microorganisms. 2020 Apr 3;8(4):510. doi: 10.3390/microorganisms8040510 (PMC7232256; doi:10.3390/microorganisms8040510)
Supplement: Supplementary file 1 [file microorganisms-08-00510-s001.pdf]

Supplementary material

Changes in the Trophic Pathways within the Microbial Food Web in the Global Warming Scenario: An Experimental Study in the Adriatic Sea

Mladen Šolić [1], Danijela Šantić [1,*], Stefanija Šestanović [1], Natalia Bojanić [1], Slaven Jozić [1], Marin Ordulj [2], Ana Vrdoljak Tomaš [1], and Grozdan Kušpilić [1]

[1] Institute of Oceanography and Fisheries, 21000 Split, Croatia; solic@izor.hr (M.Š.); sesta@izor.hr (S.Š.); bojanic@izor.hr (N.B.); sjozic@izor.hr (S.J.); ana.vrdoljak@izor.hr (A.V.T.); kuspe@izor.hr (J.K.)
[2] Department of Marine Studies, University of Split, 21000 Split, Croatia; marin.ordulj@unist.hr (M.O.)

* Correspondence: segvic@izor.hr; Tel.: 385-21-408 006

Table S1. Environmental parameter values and initial (ambient) abundances of studied plankton components.

**Environmental parameters:**	**Values**
Temperature (°C)	14.0
Salinity (psu)	34.26
Nitrate (NO_3^-) (μM)	13.44
Nitrite (NO_2^-) (μM)	7.61
Ammonium (NH_4^+) (μM)	2.23
Phosphate (PO_4^{3-}) (μM)	0.10
**Microbial abundances:**	
High nucleic-acid bacteria (HNA) (x10^6 cells mL^{-1})	0.19
Low nucleic-acid bacteria (LNA) (x10^6 cells mL^{-1})	0.26
Prochlorococcus (PROC) (x10^3 cells mL^{-1})	0.55
Synechococcus (SYN) (x10^3 cells mL^{-1})	1.96
Picoeukaryotes (PE) (x10^3 cells mL^{-1})	2.06
Heterotrophic nanoflagellates (HNF) (x10^3 cells mL^{-1})	1.01
Ciliates (CIL) (cells mL^{-1})	2.06

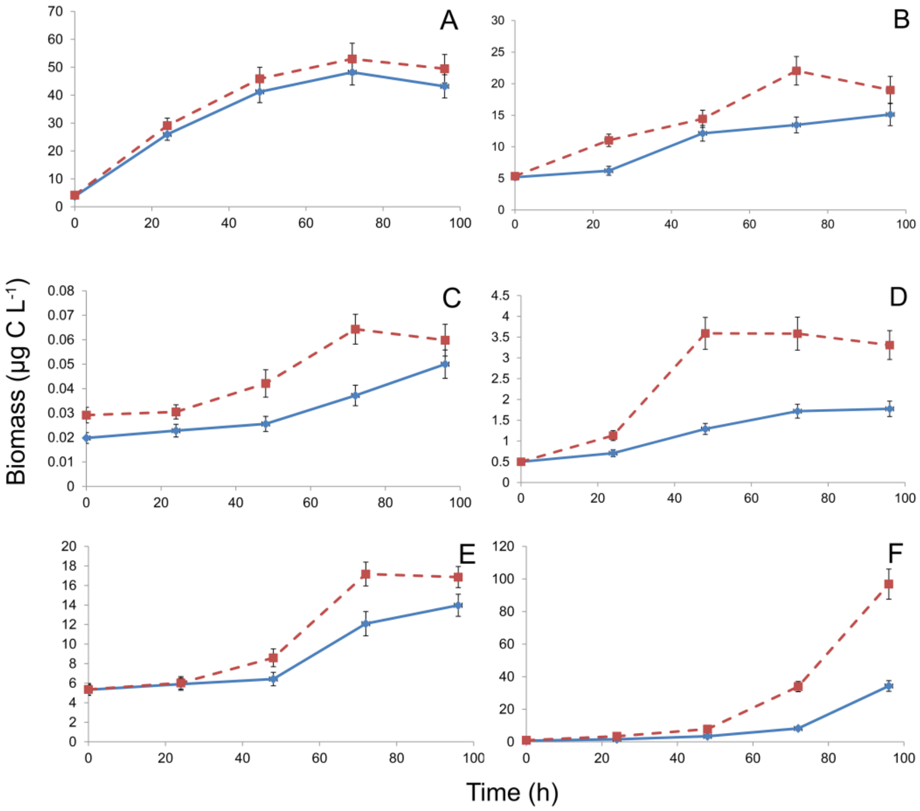

Figure S1. Biomass changes throughout experiments (A – high nucleic-acid bacteria; B - low nucleic-acid bacteria; C – *Prochlorococcus*; D – *Synechococcus*; E – picoeukaryotes; F – heterotrophic nanoflagellates). Full lines – ambient temperature; dashed lines – 3°C elevated temperature.

Table S2. Mean ± standard deviation of growth rate (μ), carrying capacity (K), growth efficiency, total grazing rate (g_{TOT}), HNF ingestion rate (I_{NF}), ciliate ingestion rate (I_{CIL}), prey standing stock removal (SSR), prey production removal (PPR) and production/grazing ratio (P/G) for the studied microbial groups (HNA – high nucleic-acid bacteria; LNA – low nucleic-acid bacteria; HB – heterotrophic bacteria = HNA+LNA; PROC – *Prochlorococcus*; SYN – *Synechococcus*; PE – picoeukaryotes; HNF – heterotrophic nanoflagellates; CIL - ciliates) at ambient temperature (T1) and 3°C elevated temperature (T2). The differences of studied parameters between two temperatures were tested with t-test for dependent samples. Percentage changes in parameter values in increased temperature conditions (T2) compared to initial ambient temperature (T1) were noted in the last column (positive changes are bolded).

PARAMETER	T1 (14°C)	T2 (17°C)	t-test p value	Change (%)
**μ (day^{-1})**				
HNA	1.197±0.130	1.210±0.135	n.s.	
LNA	0.422±0.040	0.483±0.050	<0.01	**+ 14.5**
PROC	0.249±0.025	0.371±0.040	<0.01	**+ 49.0**
SYN	0.438±0.045	1.647±0.074	<0.01	**+ 276.0**
PE	0.452±0.041	0.521±0.049	<0.01	**+ 15.3**
HNF	0.631±0.062	0.700±0.068	<0.01	**+ 10.9**
**K (cells mL^{-1})**				
HNA (x10^{6})	2.402±0.255	2.251±0.253	<0.05	- 6.7
LNA (x10^{6})	0.837±0.101	1.121±0.103	<0.01	**+ 33.9**
PROC (x10^{3})	1.507±0.162	1.782±0.170	<0.01	**+ 18.2**

SYN (x10^3)	6.94±0.705	14.193±1.553	<0.01	**+ 104.5**
PE (x10^3)	6.213±0.566	6.716±0.614	<0.01	**+ 8.1**
HNF (x10^3)	6.837±0.655	10.177±0.982	<0.01	**+ 48.9**
**Growth efficiency (%)**				
HB	41.62±3.85	22.73±2.60	<0.01	- 45.4
HNF	48.63±2.60	40.14±2.71	<0.01	- 17.5
CIL	22.55±2.45	12.26±1.25	<0.01	- 45.8
**g_{TOT} (day^{-1})**				
HNA	0.304±0.033	0.290±0.032	<0.05	- 4.6
LNA	0.074±0.007	0.032±0.003	<0.01	- 56.8
PROC	0.084±0.009	0.163±0.018	<0.01	**+ 94.0**
SYN	0.258±0.024	0.510±0.047	<0.01	**+ 97.7**
PE	0.248±0.023	0.313±0.027	<0.01	**+ 26.2**
HNF	0.109±0.011	0.119±0.012	<0.01	**+ 9.2**
**I_{NF} (pgC NF^{-1} day^{-1})**				
HNA	0.751±0.076	0.566±0.054	<0.01	- 24.6
LNA	0.081±0.007	0.056±0.004	<0.01	- 30.9
PROC	0.00054±0.00005	0.00128±0.00012	<0.01	**+ 137.0**
SYN	0.061±0.006	0.264±0.022	<0.01	**+ 332.8**
PE	0.291±0-031	0.445±0.042	<0.01	**+ 52.9**
**I_{CIL} (ngC CIL^{-1} day^{-1})**				
HNA	1.569±0.145	1.547±0.152	n.s.	
LNA	0.325±0.035	0.113±0.018	<0.01	- 65.2
PROC	0.0011±0.0001	0.0021±0.0002	<0.01	**+ 90.9**
SYN	0.106±0.013	0.063±0.006	<0.01	- 40.6
PE	1.506±0.140	1.218±0.120	<0.01	- 19.1
HNF	1.093±0.106	1.422±0.138	<0.01	**+ 31.1**
**SSR (%)**				
HNA	28.44±2.521	27.07±2.952	<0.05	- 4.84
LNA	7.31±0.840	3.20±0.366	<0.05	- 56.29
PROC	8.11±0.910	15.82±1.785	<0.05	**+ 95.20**
SYN	24.22±3.081	41.50±4.903	<0.01	**+ 71.38**
PE	23.17±2.651	29.07±2.903	<0.01	**+ 25.46**
HNF	10.39±0.983	11.28±1.254	<0.05	**+ 8.57**
**PPR (%)**				
HNA	36.36±3.570	43.44±4.461	<0.01	**+ 19.46**
LNA	21.67±2.121	24.20±2.550	<0.01	**+ 11.7**
PROC	71.99±6.758	83.28±7.906	<0.01	**+ 15.67**
SYN	63.93±5.901	33.68±3.780	<0.01	- 47.33
PE	70.56±6.907	72.29±6.916	<0.01	**+ 2.44**
HNF	3.80±0.310	17.33±1.700	<0.01	**+ 355.9**
**Production/Grazing**				
HNA	2.753±0.225	2.307±0.230	<0.01	- 19.3
LNA	4.598±0.570	4.154±0.045	<0.05	- 10.7
PROC	1.376±0.150	1.197±0.175	<0.01	- 15.0
SYN	1.575±0.178	2.973±0.345	<0.01	**+ 88.8**
PE	1.419±0.158	1.378±0.155	<0.05	- 3.0
HNF	26.504±2.905	5.764±0.672	<0.01	- 359.8

Table S2. Regression statistics for the Arrhenius plots showing the relationship of temperature (1/Kelvin x 1000) and the natural logarithm of production and grazing. E_a - values of activation energy, where R is universal gas constant (8.314 J mol^{-1} K^{-1}); R^2 – coefficient of determination; p – significance level of the regression analyses (ANOVA).

Production:	Slope (E_a/R)	R^2	*p*
HNA	2.98	0.992	<0.001
LNA	11.51	0.983	<0.001
PROC	24.82	0.958	<0.001
SYN	50.83	0.996	<0.001
PE	3.09	0.967	<0.001
HNF	9.43	0.989	<0.001
**Grazing:**			
HNA	7.91	0.878	<0.001
LNA	14.58	0.912	<0.001
PROC	28.87	0.845	<0.001
SYN	33.03	0.879	<0.001
PE	3.76	0.870	<0.001
HNF	24.86	0.856	<0.001

Table S3. Review of niche breadth and niche overlap measures

Smith's measure of niche breadth (FT)

Smith (1982) proposed this measure of niche breadth:

$$\boldsymbol{FT} = \sum_{i=1}^{n} \left(\sqrt{\boldsymbol{p_j a_j}}\right)$$

where p_j is proportion of consumed individuals of prey *j*; a_j is a proportion of prey *j* in the total prey abundances; *n* is the number of prey types. Smith's measure of niche breadth varies from 0 (minimal) to 1.0 (maximal) and thus is a standardized measure.

Hurlbert's measure of niche breadth (B')

Hurlbert's niche breadth (Hurlbert, 1978) is calculated as;

$$B' = \frac{1}{\sum \left(\frac{p_j^2}{a_j}\right)}$$

where parameters p_j and a_j have the same meaning as in Smith's equation.

Hurlbert's niche breadth is standardized to a scale of 0-1 using the equation:

$$B_S = \frac{B' - a_{min}}{1 - a_{min}}$$

where B_S is Hurlbert's standardized niche breadth; *B'* is Hurlbert's niche breadth; a_{min} is the smallest observed proportion of all the prey abundances (minimum a_j).

One way of understanding food web organization and possible competition between predators for sharing prey is the measurement of overlap in prey consumption among predators (food niche overlap). In this study we compared three different indices.

Renkonen overlap index or *percentage similarity measure*

This index proposed by Renkonen (1938) is the simplest measure of niche overlap and is given by:

$$P_{jk} = \left[\sum_{i=1}^{n} minimum\ p_{ij}, p_{ik}\right] \text{x } 100$$

where P_{jk} is percentage of overlap between predator *j* and predator *k*; p_{ij} is the proportion of prey *i* in the total prey used by predator *j*; p_{ik} is the proportion of prey *i* in the total prey used by predator *k*; *n* is the number of prey types.

Horn's index of overlap (R_o)

This index is also a similarity index based on information theory (Horn, 1966). It is calculated as follows:

$$R_o = \frac{\sum(p_{ij} + p_{ik}) \log(p_{ij} + p_{ik}) - \sum p_{ij} \log p_{ij} - \sum p_{ik} \log p_{ik}}{2 \log(2)}$$

where parameters p_{ij} and p_{ik} have the same meaning as in Renkonen's equation.

Hurlbert's index of overlap (L)

This index (Hurlbert, 1978) defines niche overlap as the degree to which the frequency of encounter between two predators is higher or lower than it would be if each predator utilized each prey type in proportion to their abundance in the environment. Thus, this index respects the fact that environmental abundance prey items are different. This index is calculated by:

$$L = \sum_{i=1}^{n} \left(\frac{p_{ij} p_{ik}}{a_i} \right)$$

where parameters p_{ij} and p_{ik} have the same meaning as in Renkonen's equation and a_i is the proportional amount of prey *i* in the environment. Unlike the first two overlap indices, which could range from 0 to 1, Hurlbert's overlap index is equal to 1.0 when both predators utilize each prey in proportion to its abundance; 0 when two predators share no prey, and >1.0 when two predators both use certain prey items more intensively than others and the preferences of two predators for prey types tend to coincide.

Table S4. Mean ± standard deviation of growth rate (μ), carrying capacity (K), growth efficiency, total grazing rate (g_{TOT}), HNF ingestion rate (I_{HNF}), ciliate ingestion rate (I_{CIL}), prey standing stock removal (SSR), prey production removal (PPR) and production/grazing ratio (P/G) for the studied microbial groups (HNA – high nucleic-acid bacteria; LNA – low nucleic-acid bacteria; HB – heterotrophic bacteria = HNA+LNA; PROC – *Prochlorococcus*; SYN – *Synechococcus*; PE – picoeukaryotes; HNF – heterotrophic nanoflagellates; CIL - ciliates) at ambient temperature (T1) and 3°C elevated temperature (T2). The differences of studied parameters between two temperatures were tested with t-test for dependent samples. Percentage changes in parameter values in increased temperature conditions (T2) compared to initial ambient temperature (T1) were noted in the last column (positive changes are bolded).

PARAMETER	T1 (14°C)	T2 (17°C)	t-test *p* value	Change (%)
**μ (day^{-1})**				
HNA	1.197±0.130	1.210±0.135	n.s.	
LNA	0.422±0.040	0.483±0.050	<0.01	**+ 14.5**
PROC	0.249±0.025	0.371±0.040	<0.01	**+ 49.0**
SYN	0.438±0.045	1.647±0.074	<0.01	**+ 276.0**
PE	0.452±0.041	0.521±0.049	<0.01	**+ 15.3**
HNF	0.631±0.062	0.700±0.068	<0.01	**+ 10.9**
**K (cells mL^{-1})**				
HNA (x10^{6})	2.402±0.255	2.251±0.253	<0.05	- 6.7
LNA (x10^{6})	0.837±0.101	1.121±0.103	<0.01	**+ 33.9**
PROC (x10^{3})	1.507±0.162	1.782±0.170	<0.01	**+ 18.2**
SYN (x10^{3})	6.94±0.705	14.193±1.553	<0.01	**+ 104.5**
PE (x10^{3})	6.213±0.566	6.716±0.614	<0.01	**+ 8.1**
HNF (x10^{3})	6.837±0.655	10.177±0.982	<0.01	**+ 48.9**
**Growth efficiency (%)**				
HB	41.62±3.85	22.73±2.60	<0.01	- 45.4
HNF	48.63±2.60	40.14±2.71	<0.01	- 17.5
CIL	22.55±2.45	12.26±1.25	<0.01	- 45.8
**g_{TOT} (day^{-1})**				
HNA	0.304±0.033	0.290±0.032	<0.05	- 4.6
LNA	0.074±0.007	0.032±0.003	<0.01	- 56.8
PROC	0.084±0.009	0.163±0.018	<0.01	**+ 94.0**
SYN	0.258±0.024	0.510±0.047	<0.01	**+ 97.7**
PE	0.248±0.023	0.313±0.027	<0.01	**+ 26.2**
HNF	0.109±0.011	0.119±0.012	<0.01	**+ 9.2**
**I_{HNF} (pgC HNF^{-1} day^{-1})**				
HNA	0.751±0.076	0.566±0.054	<0.01	- 24.6
LNA	0.081±0.007	0.056±0.004	<0.01	- 30.9
PROC	0.00054±0.00005	0.00128±0.00012	<0.01	**+ 137.0**
SYN	0.061±0.006	0.264±0.022	<0.01	**+ 332.8**

PE	0.291±0-031	0.445±0.042	<0.01	**+ 52.9**
**I_{CIL} (ngC CIL^{-1} day^{-1})**				
HNA	1.569±0.145	1.547±0.152	n.s.	
LNA	0.325±0.035	0.113±0.018	<0.01	- 65.2
PROC	0.0011±0.0001	0.0021±0.0002	<0.01	**+ 90.9**
SYN	0.106±0.013	0.063±0.006	<0.01	- 40.6
PE	1.506±0.140	1.218±0.120	<0.01	- 19.1
HNF	1.093±0.106	1.422±0.138	<0.01	**+ 31.1**
**SSR (%)**				
HNA	28.44±2.521	27.07±2.952	<0.05	- 4.84
LNA	7.31±0.840	3.20±0.366	<0.05	- 56.29
PROC	8.11±0.910	15.82±1.785	<0.05	**+ 95.20**
SYN	24.22±3.081	41.50±4.903	<0.01	**+ 71.38**
PE	23.17±2.651	29.07±2.903	<0.01	**+ 25.46**
HNF	10.39±0.983	11.28±1.254	<0.05	**+ 8.57**
**PPR (%)**				
HNA	36.36±3.570	43.44±4.461	<0.01	**+ 19.46**
LNA	21.67±2.121	24.20±2.550	<0.01	**+ 11.7**
PROC	71.99±6.758	83.28±7.906	<0.01	**+ 15.67**
SYN	63.93±5.901	33.68±3.780	<0.01	- 47.33
PE	70.56±6.907	72.29±6.916	<0.01	**+ 2.44**
HNF	3.80±0.310	17.33±1.700	<0.01	**+ 355.9**
**Production/Grazing**				
HNA	2.753±0.225	2.307±0.230	<0.01	- 19.3
LNA	4.598±0.570	4.154±0.045	<0.05	- 10.7
PROC	1.376±0.150	1.197±0.175	<0.01	- 15.0
SYN	1.575±0.178	2.973±0.345	<0.01	**+ 88.8**
PE	1.419±0.158	1.378±0.155	<0.05	- 3.0
HNF	26.504±2.905	5.764±0.672	<0.01	- 359.8
